# Epidemiology and burden of Severe Acute Respiratory Infections (SARI) in the aftermath of COVID-19 pandemic: A prospective sentinel surveillance study in a Tunisian Medical ICU, 2022/2023

**DOI:** 10.1371/journal.pone.0294960

**Published:** 2023-12-15

**Authors:** Mohamed Boussarsar, Emna Ennouri, Naima Habbachi, Nabil Bouguezzi, Khaoula Meddeb, Salma Gallas, Malek Hafdhi, Marwa Zghidi, Radhouane Toumi, Imen Ben Saida, Salma Abid, Ilhem Boutiba-Ben Boubaker, Latifa Maazaoui, Hakim El Ghord, Ahlem Gzara, Rihab Yazidi, Afif Ben Salah

**Affiliations:** 1 University of Sousse, Faculty of Medicine of Sousse, Sousse, Tunisia; 2 Medical Intensive Care Unit, Research Laboratory “Heart Failure”, Farhat Hached University Hospital, Sousse, Tunisia; 3 National Influenza Centre-Tunis, Unit Virology, Microbiology Laboratory, Charles Nicolle Hospital, Tunis, Tunisia; 4 University of Tunis El Manar, Faculty of Medicine of Tunis, Tunis, Tunisia; 5 Primary Health Care Directorate, Tunis, Tunisia; 6 Laboratory of Transmission, Control and Immunobiology of Infections (LR11IPT02), Institut Pasteur de Tunis, Tunis-Belvédère, Tunisia; 7 Service of Medical Epidemiology, Institut Pasteur de Tunis, Tunis-Belvédère, Tunisia; 8 Department of Family and Community Medicine, College of Medicine and Medical Sciences (CMMS), Arabian Gulf University (AGU), Manama, Bahrain; 9 Laboratory of Transmission, Control and Immunobiology of Infections LR16IPT02, Institut Pasteur de Tunis, University of Tunis, El Manar, Tunis, Tunisia; Taipei Medical University, TAIWAN

## Abstract

**Background:**

Severe Acute Respiratory Infections (SARI) caused by influenza and other respiratory viruses pose significant global health challenges, and the COVID-19 pandemic has further strained healthcare systems. As the focus shifts from the pandemic to other respiratory infections, assessing the epidemiology and burden of SARI is crucial for healthcare planning and resource allocation. Aim: to understand the impact of the post-pandemic period on the epidemiology of SARI cases, clinical outcomes, and healthcare resource utilization in Tunisia.

**Methods:**

This is a prospective study conducted in a Tunisian MICU part of a national sentinel surveillance system, focusing on enhanced SARI surveillance. SARI cases from week 39/2022, 26 September to week 19/2023, 13 May were included, according to a standardized case definition. Samples were collected for virological RT-PCR testing, and an electronic system ensured standardized and accurate data collection. Descriptive statistics were performed to assess epidemiology, trends, and outcomes of SARI cases, and univariate/multivariate analyses to assess factors associated with mortality.

**Results:**

Among 312 MICU patients, 164 SARI cases were identified during the study period. 64(39%) RT-PCR were returned positive for at least one pathogen, with influenza A and B strains accounting for 20.7% of cases at the early stages of the influenza season. The MICU experienced a significant peak in admissions during weeks 1-11/2023, leading to resource mobilization and the creation of a surge unit. SARI cases utilized 1664/3120 of the MICU-stay days and required 1157 mechanical ventilation days. The overall mortality rate among SARI cases was 22.6%. Age, non-COPD, and ARDS were identified as independent predictors of mortality.

**Conclusions:**

The present study identified a relatively high rate of SARI cases, with 39% positivity for at least one respiratory virus, with influenza A and B strains occurring predominantly during the early stages of the influenza season. The findings shed light on the considerable resource utilization and mortality associated with these infections, underscoring the urgency for proactive management and efficient resource allocation strategies.

## Background

Severe Acute Respiratory Infections (SARI) caused by influenza and other respiratory viruses have long been recognized as a significant public health concern, leading to substantial morbidity and mortality worldwide [1]. In the wake of the COVID-19 pandemic, the global healthcare landscape has been profoundly impacted, with an overwhelming focus on managing and understanding the novel coronavirus and its associated respiratory illness [2]. Consequently, other respiratory infections may have received less attention during this time [3].

Recently proposed integrated approaches to respiratory virus surveillance, supported by the World Health Organization (WHO), mark a significant shift from seasonal to year-round vigilance. This change involves the integration of various pathogens, diverse data sources, and multisectoral collaboration. It acknowledges that respiratory virus threats can occur throughout the year, not just seasonally, and seeks to understand the interactions between different pathogens, utilize various data sources for better decision-making, and engage multiple stakeholders to enhance global public health preparedness. These integrated strategies aim to provide a proactive, holistic, and data-driven approach to address evolving infectious disease challenges [4].

Tunisia, like many other countries, has faced unprecedented challenges in providing critical care services during the COVID-19 pandemic [5]. The strain on healthcare resources, the implementation of infection control measures, and the elevated public awareness of respiratory illnesses have had a profound influence on healthcare-seeking behaviors, healthcare delivery systems, and disease patterns [6–8]. As the COVID-19 pandemic subsides and attention shifts towards post-pandemic recovery, it is crucial to assess the implications of this crisis on other respiratory infections. Nevertheless, it is noteworthy that many National Influenza Centers (NICs) swiftly adapted to this new challenge, highlighting the significance of robust national influenza surveillance systems. These adaptative measures hold the promise of enhancing global respiratory surveillance in the future, although concerns about the sustainability of such initiatives persist [9].

Understanding the epidemiology and burden of SARI in the aftermath of the COVID-19 pandemic [10] is of utmost importance for several reasons. First, the co-circulation of influenza and other respiratory viruses and the ongoing risk of influenza outbreaks necessitates a comprehensive understanding of the impact of SARI on healthcare systems [11]. Second, the influence of the COVID-19 pandemic on SARI cases, clinical outcomes, and healthcare resource utilization needs to be evaluated to inform future pandemic preparedness strategies and resource allocation. Third, the post-pandemic period presents an opportunity to assess changes in healthcare-seeking behaviors, vaccination coverage, and healthcare infrastructure that may have long-lasting implications for SARI management.

This prospective study aims to assess the epidemiology and quantify the burden of SARI related to influenza and other respiratory viruses in a Tunisian Medical Intensive Care Unit (MICU) following the COVID-19 pandemic.

## Methods

### 1. Design

This is a prospective observational study within an enhanced SARI influenza surveillance site.

### 2. Setting

The study was conducted in the MICU of Farhat Hached University Hospital, Sousse, one of the 11 SARI sentinel centers identified as a representative sample of MICUs in Tunisia that are serving as sentinel sites for SARI surveillance [12]. The choice of this MICU relied on the geographic position in the center of the country, the 735-bed hospital capacity, and accessibility. This site is capable of providing comprehensive data on SARI cases and has the necessary infrastructure to support data collection. This is a 12-bed MICU with a large respiratory diseases recruitment with nearly 500 ICU admissions per year, 200 invasively ventilated patients, and 150 noninvasively ventilated patients per year.

### 3. Description of the surveillance system

Tunisia’s influenza surveillance system involves key stakeholders: The Primary Health Care Directorate (DSSB) coordinating the National Influenza Program, the National Influenza Center (NIC) at Charles Nicolle Hospital conducting virology testing, and a network of 113 ILI (Influenza-Like Illness) sentinel surveillance sites and 11 SARI centers. SARI sites report cases from October to April of the following year, aligning with the influenza season concerning the northern hemisphere’s cold period and migratory bird activity. This comprehensive approach has guaranteed, since 2015, timely detection and response to influenza outbreaks, thus safeguarding public health in the country [12, 13].

### 4. Population

The study included all consecutive patients admitted to the MICU with the initial diagnosis of SARI from September 26, 2022 (Week 39) to May 13, 2023 (Week 19).

### 5. Definitions

A standardized case definition for SARI based on Global epidemiological surveillance standards for influenza: World Health Organization [14].

SARI case was defined as an acute respiratory infection with a history of fever or measured fever of ≥ 38°C and cough, with onset within the past 10 days, and requiring hospitalization [14]. Based on clinical features, imaging, and PaO2/FiO2, patients were classified according to Berlin criteria for acute respiratory distress syndrome (ARDS) [15].

The term "post-pandemic period" in our context pertains to the initial influenza season (from week 39/2022, 26 September to 19/2023, 13 May) that commenced immediately following the conclusion of the last peak of the SARS-CoV-2 pandemic, which was recorded at the end of September 2022, in Tunisia.

At least one comorbidity was defined as one of the following comorbidities collected at MICU admission: hypertension, diabetes mellitus, chronic obstructive pulmonary disease, obesity hypoventilation syndrome, asthma, chronic heart failure, cancer, immune diseases, and immunosuppression.

WHO score was calculated referring to the Eastern Cooperative Oncology Group Performance Status [16] which is a score ranging from zero (“fully active”) through three (“capable of only limited self-care”) to five (“dead”) and has been adopted by the WHO and commonly used since its conception in 1982 to evaluate performance and functional status in the general population.

### 6. Sampling / Laboratory testing

SARI case definition was applied at MICU admission. All samplings were collected within 48 hours of MICU admission. The surveillance system focused only on community-acquired infections. Nasopharyngeal swabs were obtained from patients who were breathing spontaneously, while tracheal aspirates were collected from those who were invasively ventilated. Samples were placed in a viral transport medium. All samples were promptly stored at +4°C. Subsequently, they were transported to the National Influenza Center (NIC) within 72 hours, on scheduled days, three times a week. The NIC, a centralized laboratory facility, receives all of the samples to be tested for influenza, SARS-CoV-2, and other respiratory viruses, according to the national algorithm. Firstly, all samples were tested for flu A and B and SARS-CoV-2 using the United States Centers for Disease Control and Prevention (USCDC) multiplex real-time RT-PCR (Flu SC2) [17]. Then, flu A positive samples were subtyped (A(H1N1)pdm09 and A(H3N2), and lineage identification was done for flu B positive samples using the CDC protocol. In parallel, all samples were tested for multiple respiratory viruses (influenza A, influenza A(H1N1)pdm09), influenza B, coronaviruses NL63, 229E, OC43 and HKU1, parainfluenza viruses 1, 2, 3 and 4, metapneumovirus A and B, rhinovirus, respiratory syncytial viruses A and B, adenovirus, enterovirus, human bocavirus) and *Mycoplasma pneumoniae* using the FTD respiratory 21 (Siemens).

The results were communicated to the sentinel sites, the DSSB, and the WHO FluNet global monitoring system [13, 18].

### 7. Data collection

The national sentinel surveillance system designed a comprehensive data collection electronic system to capture relevant information for each SARI case, including demographic data, and underlying condition (The following comorbidities were collected: Hypertension, Diabetes mellitus, Chronic obstructive pulmonary diseases, obesity hypoventilation syndrome, asthma, chronic heart failure, cancer, immune diseases, immunosuppression), vaccination history (respectively, 2022/2023 for influenza vaccine and at least one dose for SARS-CoV-2 vaccine), delay between symptoms and MICU admission and between symptoms and sampling, clinical characteristics at MICU admission, laboratory results, and outcome measures. The data were consolidated for each patient on a standardized electronic form. The form utilized in the sentinel surveillance system underwent anonymization using a pseudonymization technique to safeguard the privacy and confidentiality of the individuals involved, aligning with ethical considerations. To avoid inconsistencies in the documentation of SARI cases or the identification of respiratory virus infections and thus ensure the accuracy of the burden estimates, two ICU residents (NH, RT) were trained to fulfill the standardized electronic forms, and two senior doctors (EE, MB) validated the forms. This is a mandatory task before it can be sent to NIC and DSSB.

### 8. Data analysis and statistics

Patients were categorized into two groups based on the results of their RT-PCR tests: the RT-PCR positive group, comprising those who tested positive for at least one respiratory virus pathogen included in the panel, and the RT-PCR negative group, comprising those who tested negative for these pathogens. Descriptive statistics were performed to characterize SARI cases epidemiological, clinical, interventional, and outcome features. Frequencies and distribution patterns of SARI cases and trends of identified viruses were assessed.

The assessment of the burden of SARI cases involved the evaluation of several key factors. Firstly, we examined the proportion and trends of SARI cases admissions within the overall MICU admissions. This analysis aimed to detect peak periods with the highest burden of SARI cases (surge unit, nurses’ redeployment, material, and organizational considerations). Secondly, we analyzed the length of stay for SARI cases in the MICU. Thirdly, we assessed the number of invasive mechanical ventilation (IMV) and noninvasive ventilation (NIV) days. Lastly, we investigated the compared mortality rates, respectively among SARI cases, non-SARI cases in the same period, and MICU total admissions in the years 2022 and 2019.

Patient characteristics were described as frequencies and percentages for categorical variables, means and standard deviation for normal continuous variables, and medians and interquartile ranges for non-normal continuous variables. Data distribution was assessed using the Shapiro-Wilk test and histogram analysis. Chi-square or Fischer’s exact test was used to compare categorical variables and Student’s *t*-test or Mann-Whitney U test to compare continuous variables. Statistical significance was set at p < 0.05.

Statistical analyses were performed using SPSS18 software.

Student’s t and Mann–Whitney U tests were used when dealing with normally and non-normally distributed continuous variables. Chi-squared and Fisher’s exact tests were used with categorical variables depending on the sample size and the expected cell frequencies.

Univariate and multivariate analyses were performed to identify factors independently associated with ICU mortality. For the multivariate analysis: Despite a rather small sample size, we used a binary logistic regression multivariate analysis, as the number of variables included in the multivariate model is much smaller than the number of patients in the mortality event group. Binary logistic regression multivariate analysis used manual stepwise backward elimination, after including variables showing a significant association with mortality in the univariate model, variables with a p-value <0.2 along variables that are expected to be associated, although it did not show significant associations in the univariate analysis.

The present observational study complied with the Reporting of Observational Studies in Epidemiology (STROBE) [19] (S1 Checklist).

### 9. Ethics

#### Humans ethics statement

All procedures performed in the present prospective study, involving human participants, were in accordance with the ethical standards of the Institutional Research and Ethics Committee of the University Hospital Farhat Hached, Sousse (IORG 0007439), the National Research Committee, and with the 1964 Helsinki Declaration and its later amendments or comparable ethical standards.

#### Adult consent to participate written

This study received approval from the Institutional Research and Ethics Committee of the University Hospital Farhat Hached, Sousse (IORG 0007439,00260022022). The need for written informed consent was waived since nasopharyngeal swabs for influenza and other viruses are considered a routine diagnostic procedure in managing SARI patients. Furthermore, no other interventional procedures were conducted in the present study. Data were anonymized upon collection, and the authors did not have access to identifying information.

#### Human accordance statement

All procedures were carried out in strict compliance with the guidelines set by the Institutional Research and Ethics Committee of the University Hospital Farhat Hached, Sousse, and the National Research Committee. The study also adhered to the principles outlined in the 1964 Helsinki Declaration and its subsequent amendments or comparable ethical guidelines.

## Results

### Epidemiology of SARI cases

The flow of MICU-admitted patients and SARI cases was distributed as follows: 312 patients were admitted during the study period, which spanned from week 39/2022 to week 19/2023. Among these patients, 164 (52.6%) were identified as SARI cases, RT-PCR positive for at least one respiratory virus, 64 (39%) and RT-PCR negative, 100 (61%).

In the provided figure, the pink area represents the influenza season period spanning from week 39/2022 to week 19/2023. Notably, a substantial peak in MICU admissions, predominantly comprising SARI cases, is observed between week 1 and week 11 of 2023 (indicated by the dashed lines). During this critical period, a surge in capacity was implemented, expanding the MICU with an additional 5-bed unit to accommodate the increased patient load. Moreover, the yellow line in the graph marks the ILI epidemiological threshold identified within the ILI sentinel surveillance sites in Tunisia [20]. Remarkably, this threshold is observed one week after the significant shift in SARI cases’ ICU admissions, which surpassed the 50% mark.

### SARI cases characteristics

Table 1 presents a comparison of patient characteristics between RT-PCR positive and negative cases, focusing on clinical, therapeutic, and outcome features. The results of the univariate analysis indicated that older age, but not the underlying condition, was associated with positive RT-PCR results. The SARI cases included in the study had a notably low rate of vaccination, respectively 40.9% against SARS-CoV-2 (at least one dose) and 9.1% against influenza. In terms of clinical presentation, ARDS was associated rather with positive RT-PCR. Among the RT-PCR negative group, out of the 38 (38%) patients who received invasive ventilation, only 7 (18.4%) tracheal aspirates identified the following pathogens: 4 cases of *Klebsiella pneumonia* and 3 cases of *Acinetobacter baumannii*.

**Table 1:** Compared clinical, therapeutic, and outcome characteristics between RT-PCR positive to at least one respiratory pathogen, and RT-PCR negative patients, among SARI cases during the influenza season 2022/2023.

Variables	Overall	RT-PCR Positive	RT-PCR Negative	P
	(N = 164)	(N = 64)	(N = 100)	
**Demographics**				
Age, median [IQR]	65 [52.25–72]	66 [57.25–72.75]	63.5 [42.5–70]	0.079
Age, [range]	[15–94]	[18–94]	[15–88]	
Sex, Male	107 (61.7)	41 (64.1)	66 (61.7)	0.867
Smoking	67 (40.9)	27 (42.2)	40 (40)	0.780
**Underlying condition** (n = 164)				
Hypertension	63 (38.4)	24 (37.5)	39 (39)	0.847
Diabetes	39 (23.8)	13 (20.3)	26 (26)	0.460
COPD	74 (45.1)	30 (46.9)	44 (44)	0.750
Asthma	6 (3.7)	3 (4.7)	3 (3)	0.679
Heart Failure	21 (12.8)	8 (12.5)	13 (13)	0.926
At least one comorbidity	145 (88.4)	57 (89.1)	88 (88)	0.836
WHO score	2 [1–3]	2 [1–3]	2 [1–3]	0.602
**Vaccination status**				
SARS-CoV-2 (at least one dose)	67 (40.9)	19 (29.7)	48 (48)	**0.023**
Influenza 2022/2023	15 (9.1)	6 (9.4)	9 (9)	0.935
**Clinical presentation at MICU admission** (n = 164)				
**Delay from symptoms to RT-PCR**	7 [5–11]	7 [4–10]	8 [5–13]	**0.043**
PaO2/FiO2	246.8±97	237.7±82.6	252.7±105.5	0.335
Radiological opacities	96 (58.5)	41 (64.1)	55 (55)	0.261
ARDS	22 (13.4)	14 (21.9)	8 (8)	**0.017**
SAPS II	32.8±11.8	32.3±9.9	33.2±12.8	0.628
**Diagnosis at MICU** (n = 164)				
COPD exacerbation	93 (56.7)	38 (59.4)	55 (55)	0.630
Pneumonia	26 (15.9)	15 (23.4)	11 (11)	**0.047**
Cardiac decompensation	10 (6.1)	2 (3.1)	8 (8)	0.318
Miscellaneous	35 (21.3)	9 (14.1)	26 (26)	0.080
**Therapeutic management** (n = 164)				
HFNC	39 (23.8)	18 (28.1)	21 (21)	0.352
NIV	111 (67.7)	49 (76.6)	62 (62)	0.085
IMV	63 (38.4)	25 (39.1)	38 (38)	0.891
Corticosteroids	95 (57.9)	42 (65.6)	53 (53)	0.103
Antibiotics	68 (41.5)	27 (42.2)	41 (41)	0.880
**Outcomes** (n = 164)				
MICU LOS	7 [5–12]	7 [4–10.75]	8 [5–13]	0.276
MICU Mortality	37 (22.6)	17 (26.6)	20 (20)	0.347

Data were expressed as n (%) for categorical variables and as median[IQR] or mean±SD for continuous variables.

COPD, Chronic Obstructive Pulmonary Disease, WHO, World Health Organization, SARS-CoV-2, PaO2/FiO2, Arterial partial pressure of oxygen/Inspired fraction of oxygen, ARDS, Acute Respiratory Distress Syndrome, SAPS II, Simplified Acute Physiology Score II, HFNC, High Flow Nasal Cannula, NIV, Non-Invasive Ventilation, IMV, Invasive Mechanical Ventilation, MICU, Medical Intensive Care Unit, LOS, Length of Stay.

### Pathogen distribution among SARI cases

**Fig 1:**
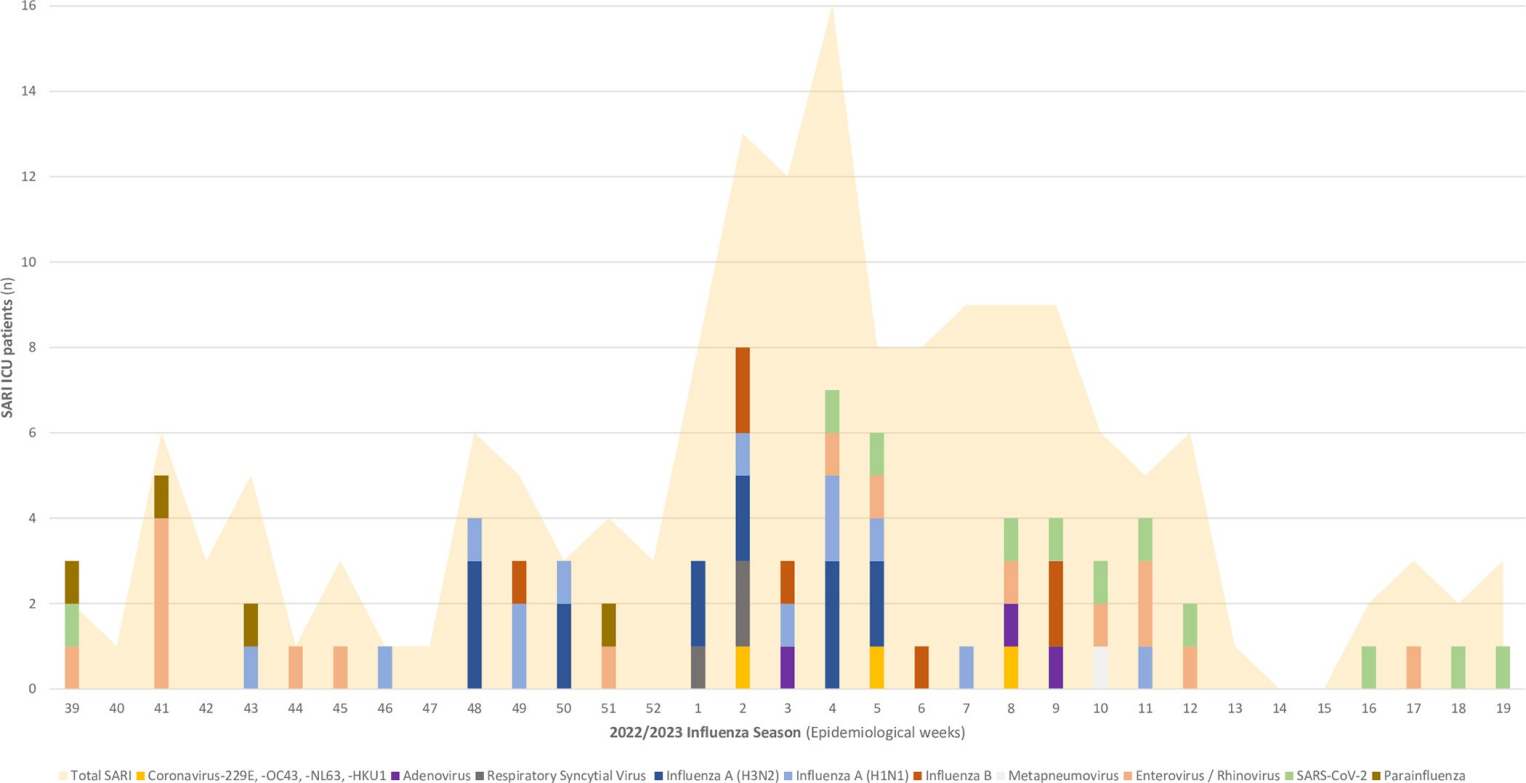
Distribution of SARI cases and trends of identified viruses using RT-PCR from week 39/2022 to week 19/2023 in the Medical Intensive Care Unit (MICU) of Farhat Hached University Hospital.

Fig 1 depicts the distribution of SARI cases throughout the study period, as well as the weekly trends of identified viruses using RT-PCR from week 39/2022 to week 19/2023 in the MICU of Frarhat Hached University Hospital. Fig 1 reveals a significant peak observed between week 1 and week 11/2023. Among the identified viruses, influenza A and B strains accounted for 20.7% of the cases, while Enterovirus/Rhinovirus represented 9.8% (Table 2). Within influenza viruses, the different strains were distributed as follows: A(H1N1), 38%, A(H3N2), 41%, B, 21%. In the first stages of the influenza season, influenza A and B strains were the predominant viruses. However, it is worth noting that towards the end of the influenza season, there was a resurgence of 11 (6.7%) cases of SARS-CoV-2 and other viruses.

**Table 2:** RT-PCR results of nasal swabs and tracheal aspirates in MICU-admitted SARI patients from week 39/2022 to week 19/2023.

	n (%)
Negative	100 (61)
Coronavirus-229E, -OC43, -NL63, -HKU1	3 (1.8)
Adenovirus	3 (1.8)
Respiratory Syncytial Virus	3 (1.8)
Bocavirus	0
Influenza A(H3N2)	14 (8.5)
Influenza A(H1N1)	13 (7.9)
Influenza B Virus	7 (4.3)
Metapneumovirus	1 (0.6)
Enterovirus / Rhinovirus	16 (9.8)
Coronavirus MERS-CoV	0
SARS-CoV-2	11 (6.7)
Parainfluenza	4 (2.4)
Coinfection	11 (6.7)

The figure reveals a significant peak of SARI cases observed between week 1 and week 11/2023. Influenza A and B strains were predominant at the beginning of the peak and SARS-CoV-2 towards the end.

### The burden of SARI cases

#### MICU workload and surge

**Fig 2:**
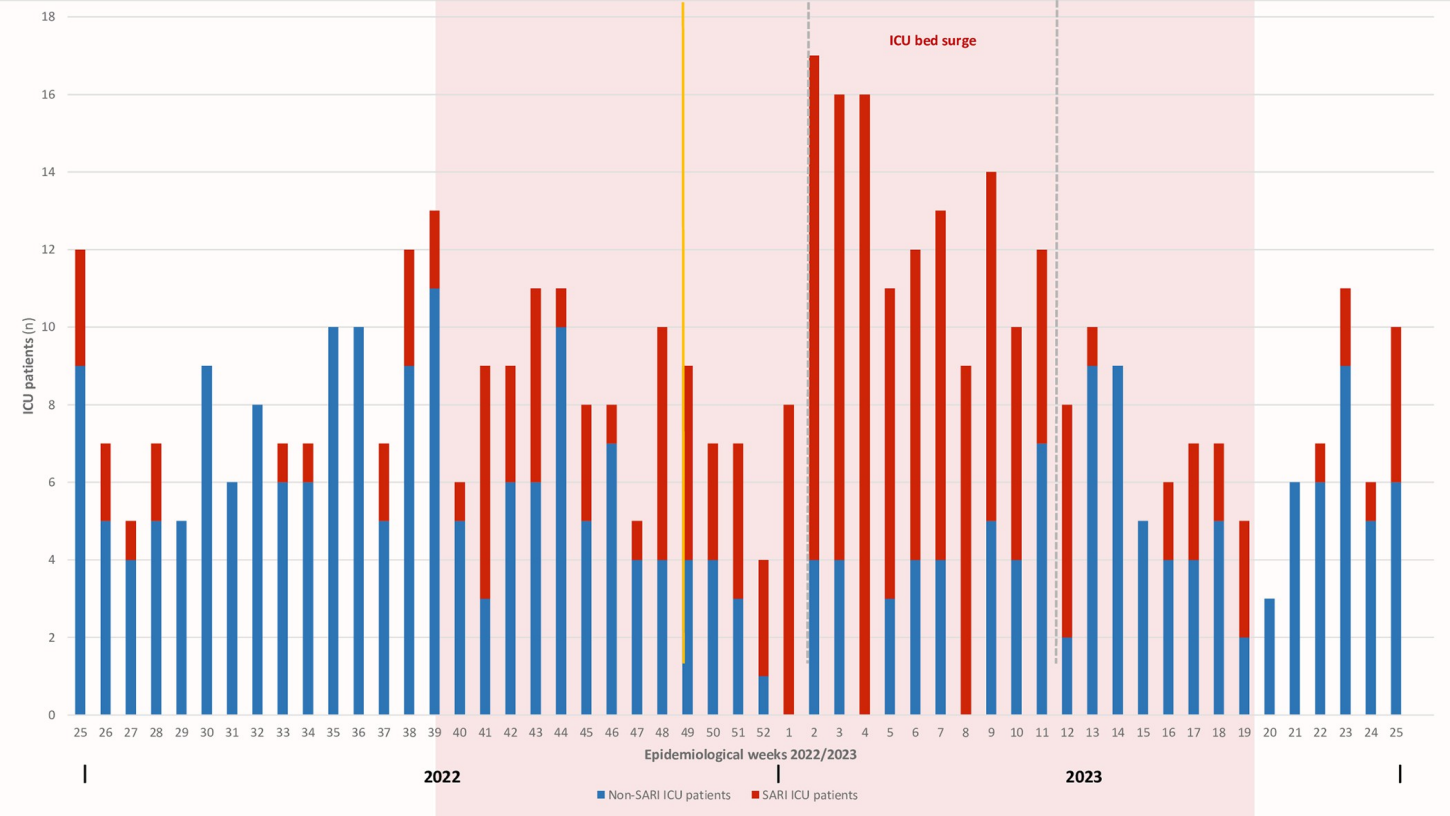
Weekly trends of MICU admissions and SARI cases from week 25/2022 to week 25/2023.

Fig 2 summarizes the weekly trends of MICU admissions and SARI cases from week 25/2022 to week 25/2023. A significant peak in MICU admissions, including a high proportion of SARI cases, was observed between week 1/2023 and week 11/2023. To manage the influx of patients during this period, a 5-bed unit was surged. Five nurses were redeployed from different medical wards to staff the surge unit, and three additional temporary work contract nurses were recruited to ensure smooth operation. As these healthcare workers had limited skills in intensive care patients’ management and monitoring, they were assisted alongside critical care residents. This placed a significant workload on healthcare providers, necessitating increased utilization of medical resources, medications, personal protective equipment (PPE), and organizational adjustments.

#### SARI cases outcomes

During the influenza season period, the SARI cases accounted for 1664 out of the total of 3120 MICU-stay days. Among these SARI cases, mechanical ventilation was required for 604 IMV days, and 553 NIV days.

In terms of mortality, the overall mortality rate among SARI cases stood at 22.6%. During the same influenza season period, non-SARI patients had a 15.3% mortality rate (p = 0.104). This figure is closely in line with the general patient MICU admission rate in 2019 (21%) and in 2022 (21.39%).

#### Risk factors associated with mortality in SARI cases

Table 3 presents the results of the univariate analysis examining factors associated with mortality among SARI cases. The factors identified in the univariate analysis were further included in a multivariate analysis to determine independent predictors of mortality. Three factors were found to be independently associated with mortality among SARI cases: (OR, [CI95%], p), age (1.028, [1.001–1.056], 0.038), COPD (0.253, [0.093–0.687], 0.007), and ARDS (19.135, [5.783–63.317], < 0.000).

**Table 3:** Univariate analysis examining factors associated with MICU mortality among SARI cases during the influenza season 2022/2023.

	Deaths	Survivors	OR	CI95%	P
	N = 38	N = 126			
Underlying condition (n = 164)					
Age	60.4±20.3	60.0±17.2	1.001	[0.981–1.022]	0.907
Hypertension	18 (47.4)	45 (35.7)	1.620	[0.778–3.374]	0.195
Diabetes	12 (31.6)	27 (21.4)	1.692	[0.756–3.787]	0.198
COPD	8 (21.1)	66 (52.4)	0.242	[0.103–0.570]	0.001
WHO score	2.0±1.2	1.97±1.05	1.027	[0.735–1.435]	0.876
Influenza vaccine	4 (10.5)	11 (8.7)	1.230	[0.368–4.111]	0.751
Positive RT-PCR (n = 164)	17 (44.7)	47 (37.3)	1.361	[0.653–2.836]	0.410
Influenza A (n = 164)	10 (26.3)	17 (13.5)	2.290	[0.945–5.546]	0.062
Peak period, (Week1-11/2023)	23 (60.5)	80 (63.5)	0.882	[0.419–1.857]	0.740
Clinical presentation at MICU admission (n = 164)					
PaO2/FiO2	189.3±87.6	264.3±93.3	0.989	[0.984–0.995]	0.000
Chest X-ray consolidations	32 (84.2)	64 (50.8)	5.167	[2.020–13.217]	0.000
ARDS	17 (44.7)	5 (4.0)	19.590	[6.524–58.825]	0.000
SAPS II	37.8±15.4	31.3±9.9	1.046	[1.013–1.079]	0.002

Data were expressed as n (%) for categorical variables and as median[IQR] or mean±SD for continuous variables.

CI, Confidence Interval, COPD, Chronic Obstructive Pulmonary Disease, WHO, World Health Organization, MICU, Medical Intensive Care Unit, PaO2/FiO2, Arterial partial pressure of oxygen/Inspired fraction of oxygen, ARDS, Acute Respiratory Distress Syndrome, SAPS II, Simplified Acute Physiology Score II.

## Discussion

As we transition into the post-COVID-19 pandemic era, understanding the burden and impact of SARI, a highly contagious respiratory infection challenging healthcare systems worldwide, remains crucial for effective healthcare planning and resource allocation [21, 22]. This prospective study aimed to shed light on the epidemiology and burden of SARI cases within a MICU during the first influenza season in the post-COVID-19 pandemic period [10]. By examining the frequency of SARI cases, the prevalence of different viral strains, resource mobilization, and mortality rates, this study provides valuable insights that can inform targeted interventions, resource allocation strategies, and evidence-based public health policies to mitigate the impact of SARI in the current healthcare landscape.

The present study demonstrated a high frequency of SARI cases among the MICU admissions within this first influenza season in the post-COVID-19 pandemic period. Influenza A accounted for the most retrieved viruses at the early stages of the influenza season. This epidemic generated high resource mobilization and relatively high mortality rates.

### Strengths and limitations

The longitudinal prospective design allowed us to establish temporal relationships and observe changes in SARI cases, clinical outcomes, and resource utilization following the COVID-19 pandemic. This provides a better understanding of the impact and trends. The specific aims of the present study constitute a targeted approach that could help gain insights into the effects of the pandemic on SARI cases and healthcare resources.

All biases are addressed and controlled. As for selection bias, and to capture the full spectrum of severity of SARI cases, all consecutive patients with SARI cases were included during the whole studied influenza season. As for information bias and to avoid inconsistencies in the documentation of SARI cases or the identification of respiratory virus infections, thus ensuring the accuracy of the burden estimates, controlled fulfillment and validation of the standardized electronic forms was ensured. For Bias related to data collection methods, to prevent introducing bias in the identification and classification of SARI cases and influenza and other respiratory viruses, the sentinel surveillance system allowed quality control over data collection methods, ensuring standardized and accurate data collection techniques. This enhanced the reliability and validity of the study’s findings, especially as exhaustive and consecutive SARI cases were included.

Several limitations could be addressed. As for generalizability, the findings of this study may be limited to the specific context of a Tunisian MICU and may not be directly applicable to other settings or populations. However, the enhanced surveillance system described herein would be useful for any system to allow assessment of the impact of SARI on the local ICU capacity to face such epidemics. Additionally, the purpose of the present study is to identify the geographical peculiarities to implement locally adapted interventions and inform public health policies to mitigate the impact of SARI. The study aims to understand the impact of the COVID-19 pandemic on SARI cases, however, external factors such as the temporal relationship between the COVID-19 pandemic and SARI cases, changes in healthcare policies, vaccination campaigns, or population behavior may also have influenced the results [23]. It may be challenging to separate the specific effects of the pandemic from other concurrent factors, but one could ask to which extent all of these factors could be, at least in part, the consequence of the pandemic.

### Results interpretation

#### Epidemiology of SARI cases

The present study revealed a significantly higher incidence of SARI cases during the 2022/23 influenza season, in comparison to the preceding 2020/2021 and 2021/2022 seasons, which coincided with the ongoing COVID-19 pandemic [18]. As in many other countries, Tunisia detected few amounts of other respiratory viruses among SARI cases during the COVID-19 pandemic [3, 8, 11, 24–26]. Several factors can potentially explain the resurgence of these respiratory viruses. Firstly, the implementation of social distancing measures to curb COVID-19 transmission may have played a role, albeit partially [27]. Secondly, the viral interference and competitive advantages of SARS-CoV-2 in modulating host immunity could be influencing this phenomenon [28]. Thirdly, the low vaccine coverage rate could be a contributing factor. Additionally, the decline in influenza immunity throughout the pandemic period could have contributed to this trend [28]. Lastly, it is worth noting that the improved data collection within the post-pandemic period, through the sentinel surveillance system, could also explain the observed high SARI cases incidence [18].

The ILI epidemiological threshold was identified at week 49 within the ILI sentinel surveillance sites in Tunisia [20]. Interestingly, this threshold aligns with the trends observed in SARI admissions in the MICU, where SARI cases surpassed half of the MICU admissions even since week 48. This correlation underscores the significance of SARI surveillance, as it demonstrates a clear lead time over medically attended ILI in the general population. The research conducted by van Asten et al. highlights the potential benefits of integrating SARI surveillance alongside ILI surveillance [29]. By monitoring SARI cases, healthcare authorities can gain valuable insights into the season-specific burden of SARI. Moreover, the early escalation of SARI cases (more than 50% of MICU admissions in the present study) can serve as an essential early warning signal, providing critical information on influenza activity and its severity [30, 31].

#### Pathogen distribution among SARI cases

In the present study, the positivity rate (for at least one respiratory virus) was observed to be 39%. Radhi et al., Chard et al., and Mohamed et al., found respectively lower positivity rates (32.5%, 12.1%, 30.8%) [32–34], although, these studies do not fully encompass all the characteristics of the present study. However, it is important to explore the reasons behind the negative results obtained from the RT-PCR tests. Several factors may contribute to this phenomenon. Firstly, it is crucial to consider the issue of pre-test probability, which is determined by the attending physicians and the definition used, as well as the completeness of its various components [35, 36]. This can impact the likelihood of obtaining negative results. Secondly, it is worth considering that the delay between the onset of symptoms and the time of sampling may have contributed, at least partially, to the RT-PCR negativity. This is supported by the significant association observed between the delay and RT-PCR results. Additionally, the negativity could potentially be attributed to the presence of other unidentified pathogens. This is especially relevant for spontaneously breathing patients who were not sampled for bacteria identification. Furthermore, it is worth noting that the yield of bacteriological exams in invasively ventilated patients is generally poor. Additionally, the accuracy and interpretation of the RT-PCR tests themselves may also contribute to the observed negative results [37]. Lastly, considering that a substantial proportion of the included patients were admitted due to acute exacerbation of COPD, it is important to acknowledge that many of them might have experienced non-viral triggers for their exacerbations. Factors like stress, anxiety, medications, physical exertion, or the natural progression of the disease could contribute to negative results in RT-PCR tests, especially since the symptoms of acute exacerbation of COPD often overlap with those outlined in the SARI definition.

The present study revealed a relatively high proportion of influenza A(H1N1), A(H3N2), and influenza B (approximately 20%), alongside enterovirus/rhinovirus (9.8%) and SARS-CoV-2 (6.7%) in SARI cases. In Tunisia, the 2022/2023 influenza season has been marked by an early onset and intense activity, with a positivity rate for at least one respiratory virus, of 58% (ILI, 70%; SARI, 54%) for at least one respiratory virus as early as week 40 [38]. There has been circulation of 19 respiratory pathogens at different levels, with a predominance of influenza A virus. The prevalence of Enterovirus/Rhinovirus has been notable both at the beginning and the end of the season. The season from week 40, 2022, to week 17, 2023, was further characterized by a triple epidemic (influenza, RSV, and SARS-CoV-2), high influenza activity, and significant RSV circulation compared to the two previous seasons [38]. In comparison to the national sentinel surveillance data, the distribution of influenza strains among MICU SARI patients was as follows: A(H1N1), 44% vs. 38%, A(H3N2), 23% vs. 41%, B, 33% vs. 21% [38]. These findings underscore the high proportion of A(H3N2) accounting for severe influenza A-related SARI among the MICU admitted patients.

Despite the use of standardized and centralized RT-PCR in the sentinel surveillance system, caution is advised when interpreting the proportions of influenza A and its subtypes, influenza B, and other respiratory viruses. Several factors should be considered, including temporal patterns, particularly in the post-pandemic period of COVID-19, dominant strains, interactions between viruses, and vaccination coverage. In the temperate zone of the northern hemisphere, the 2022–2023 influenza season exhibited distinct patterns of viral circulation and activity [39]. In October, influenza activity began to rise steadily, reaching its peak levels in December. Subsequently, between January and mid-February 2023, influenza activity decreased across most countries. Throughout this period, influenza A(H3N2) viruses were the predominant strain detected. In northern Africa, A(H3N2) viruses were prevalent from September to November, followed by a majority of A(H1N1)pdm09 detections in December and January 2023. Northern African countries and all regions in Europe reported an increasing proportion of influenza B virus detections [39].

Regarding seasonal variability, it is worth noting that both influenza A and B viruses were represented in equal proportions. This may be attributed to the reduced population immunity during the COVID-19 pandemic period, which typically influences the proportions of these viruses [40]. The circulation of other identified respiratory viruses may have been influenced by interactions and competition between viruses [41–43]. Viral interference or competitive advantages, including immune responses triggered by previous infections or vaccinations, can affect the proportions of these viruses detected in SARI cases. Compared to the literature, the Tunisian population exhibited reluctance towards influenza vaccination following the multiple COVID-19 vaccinations [44]. Compared 2022 vaccination rates were respectively, in SARI patients’ vs Tunisian general population, for influenza (9.1% vs 2.67%) and for SARS-CoV-2, at least one dose (40.9% vs 87.3%) [20, 45]. The low influenza vaccine coverage in this context could have contributed to the diverse array of influenza virus strains identified in the present study.

#### The burden of SARI cases

During weeks 51 and 52 of the 2022 influenza season, the 12-bed MICU faced a significant daily influx of SARI cases. In response, MICU bed capacity was expanded with an additional 5-surge-bed unit. This rapid expansion allowed the unit to effectively accommodate the increased number of patients. It is noteworthy to highlight the ICU team’s responsiveness and the hospital’s ability to promptly achieve the surge capacity within a short timeframe. This was facilitated by the team’s previous experience in managing similar surges during different peaks of the COVID-19 pandemic [5].

During the influenza season period, a significant proportion of the MICU resources was utilized by SARI cases, accounting for more than half of the total MICU stay days. These SARI cases required close to two-thirds of the mechanical ventilation days, resulting in a substantial impact on healthcare resources, including materials, medications, PPE, and organizational operations. The high demand for MICU-stay days and mechanical ventilation highlights the severity of the SARI cases during the influenza season. This, in turn, placed a considerable strain on the healthcare system, necessitating increased allocations of resources and manpower to address the influx of patients.

In terms of mortality, the overall mortality rate among SARI cases stood at 22.6%. During the same influenza season period, non-SARI patients had a 15.3% mortality rate. This figure is closely in line with the general patient MICU admission rate in 2019 (21%) and in 2022 (21.39%). Different rates of mortality among SARI patients were reported ranging from 4.3% to 31.7% [32, 34, 46, 47]. There was also no difference in mortality according to different strains of identified pathogens (Influenza A versus other SARI cases). Although underlying conditions were not identified as a risk factor for mortality in the present studied SARI cases, age and severity of the presentation at MICU admission were identified as the only associated factors. The “protective” effect of AE/COPD, could be attributed to the fact that those patients generally exhibit a more favorable prognosis when compared to those with conditions such as ARDS, pneumonia, acute heart failure, shock, and neurological disorders. The MICU-admitted COPD patients with the more challenging prognosis are those presenting with ARDS rather than AE/COPD triggered by upper airway infection. The identification of these factors underscores the importance of early recognition and targeted interventions for this vulnerable population.

### Future directions

Several lessons can be drawn from the results of the present study assessing the epidemiology and burden of SARI cases in the aftermath of the COVID-19 pandemic. By combining SARI and ILI surveillance data, public health practitioners can develop a more comprehensive and proactive approach to tackling influenza and other respiratory virus outbreaks. This integrated strategy empowers them to implement timely and targeted interventions, effectively mitigating the impact of influenza and other respiratory viruses on vulnerable populations and the healthcare system. In a public health crisis such as the influenza epidemic, prompt priority should be given to the creation of adapted surge ICU beds in each hospital to manage the high influx of SARI cases with its myriad of severity presentations. By understanding the impact of SARI cases on MICU resources during the influenza season, healthcare institutions can better prepare and allocate resources to ensure optimal patient care and staff safety during future outbreaks [48]. Implementing strategies to cope with increased SARI admissions can help enhance the overall resilience and responsiveness of the healthcare system in the face of influenza, and other respiratory virus, related pressures.

## Conclusions

The study revealed a high frequency of SARI cases among MICU admissions during the 2022/2023 influenza season, with influenza A being the most prevalent viral strain at the early stages of the influenza season. The epidemic generated significant resource mobilization and relatively high mortality rates. The longitudinal prospective design of the study allowed for the observation of temporal relationships and changes in SARI cases and clinical outcomes following the COVID-19 pandemic, providing valuable insights into the impact and trends.

Furthermore, the study demonstrated the necessity of prompt action and resource mobilization during peak periods of SARI cases, as evidenced by the expansion of the MICU bed capacity in response to the increased influx of patients. The adaptability and resourcefulness of healthcare professionals were crucial in managing the surge in demand for critical care services during the influenza season. Integrating SARI and ILI surveillance data can strengthen the preparedness and response to influenza and other respiratory virus outbreaks, and the creation of adapted surge ICU beds in each hospital can help manage the high influx of SARI cases effectively.

## Supporting information

S1 Checklist STROBE statement—checklist. (DOC)
