# Supplementary material for: Epidemiology and burden of Severe Acute Respiratory Infections (SARI) in the aftermath of COVID-19 pandemic: A prospective sentinel surveillance study in a Tunisian Medical ICU, 2022/2023
Source: PLoS One. 2023 Dec 15;18(12):e0294960. doi: 10.1371/journal.pone.0294960 (PMC10723666; doi:10.1371/journal.pone.0294960)
Supplement: S1 Checklist — (DOC) [file pone.0294960.s001.doc]

STROBE Statement—Checklist

**Epidemiology and Burden of Severe Acute Respiratory Infections (SARI) in the Aftermath of COVID-19 Pandemic: A Prospective Sentinel Surveillance Study in a Tunisian Medical ICU, 2022/2023.**

PLOS ONE manuscript number: PONE-D-23-24210

|  | Item No | Recommendation | Page, Line |
| --- | --- | --- | --- |
| **Title and abstract** | 1 | (*a*) Indicate the study’s design with a commonly used term in the title or the abstract | 1, 3 |
|  |  | (*b*) Provide in the abstract an informative and balanced summary of what was done and what was found | 3 |
| Introduction | | |  |
| Background/rationale | 2 | Explain the scientific background and rationale for the investigation being reported | 4, 71 |
| Objectives | 3 | State specific objectives, including any prespecified hypotheses | 5, 94 |
| Methods | | |  |
| Study design | 4 | Present key elements of study design early in the paper | 5, 100 |
| Setting | 5 | Describe the setting, locations, and relevant dates, including periods of recruitment, exposure, follow-up, and data collection | 5, 102 |
| Participants | 6 | (*a*) Give the eligibility criteria, and the sources and methods of selection of participants. Describe methods of follow-up | 6, 120 |
|  |  | (*b*) For matched studies, give matching criteria and number of exposed and unexposed | NA |
| Variables | 7 | Clearly define all outcomes, exposures, predictors, potential confounders, and effect modifiers. Give diagnostic criteria, if applicable | 6, 123  6, 169 |
| Data sources/ measurement | 8* | For each variable of interest, give sources of data and details of methods of assessment (measurement). Describe comparability of assessment methods if there is more than one group | 6, 169 |
| Bias | 9 | Describe any efforts to address potential sources of bias | 6, 156 |
| Study size | 10 | Explain how the study size was arrived at | NA |
| Quantitative variables | 11 | Explain how quantitative variables were handled in the analyses. If applicable, describe which groupings were chosen and why | 6, 170 |
| Statistical methods | 12 | (*a*) Describe all statistical methods, including those used to control for confounding | 6, 169 |
|  |  | (*b*) Describe any methods used to examine subgroups and interactions | 6, 169 |
|  |  | (*c*) Explain how missing data were addressed | NA |
|  |  | (*d*) If applicable, explain how loss to follow-up was addressed | NA |
|  |  | (*e*) Describe any sensitivity analyses | NA |
| Results | | |  |
| Participants | 13* | (a) Report numbers of individuals at each stage of study—eg numbers potentially eligible, examined for eligibility, confirmed eligible, included in the study, completing follow-up, and analysed | 10, 219 |
|  |  | (b) Give reasons for non-participation at each stage | NA |
|  |  | (c) Consider use of a flow diagram | 11, 226 |
| Descriptive data | 14* | (a) Give characteristics of study participants (eg demographic, clinical, social) and information on exposures and potential confounders | 11, 229 |
|  |  | (b) Indicate number of participants with missing data for each variable of interest | 11, 240 |
|  |  | (c) Summarise follow-up time (eg, average and total amount) | 13, 260 |
| Outcome data | 15* | Report numbers of outcome events or summary measures over time | 13, 260  15, 282 |
| Main results | 16 | (*a*) Give unadjusted estimates and, if applicable, confounder-adjusted estimates and their precision (eg, 95% confidence interval). Make clear which confounders were adjusted for and why they were included | 16, 303 |
|  |  | (*b*) Report category boundaries when continuous variables were categorized | NA |
|  |  | (*c*) If relevant, consider translating estimates of relative risk into absolute risk for a meaningful time period | NA |
| Other analyses | 17 | Report other analyses done—eg analyses of subgroups and interactions, and sensitivity analyses | NA |
| Discussion | | |  |
| Key results | 18 | Summarise key results with reference to study objectives | 17, 321 |
| Limitations | 19 | Discuss limitations of the study, taking into account sources of potential bias or imprecision. Discuss both direction and magnitude of any potential bias | 18, 347 |
| Interpretation | 20 | Give a cautious overall interpretation of results considering objectives, limitations, multiplicity of analyses, results from similar studies, and other relevant evidence | 18, 358 |
| Generalisability | 21 | Discuss the generalisability (external validity) of the study results | 18, 347 |
| Other information | | |  |
| Funding | 22 | Give the source of funding and the role of the funders for the present study and, if applicable, for the original study on which the present article is based | NA |
